# An Energy Efficient Cooperative Hierarchical MIMO Clustering Scheme for Wireless Sensor Networks

**DOI:** 10.3390/s120100092

**Published:** 2011-12-22

**Authors:** Mehwish Nasim, Saad Qaisar, Sungyoung Lee

**Affiliations:** 1 School of Electrical Engineering and Computer Science, National University of Sciences and Technology, H-12, Islamabad, Pakistan; E-Mails: mehwish.nasim@seecs.edu.pk (M.N.); saad.qaisar@seecs.edu.pk (S.Q.); 2 Department of Computer Engineering, College of Electronics and Information, Kyung Hee University, Yongin-Si 446-701, Korea

**Keywords:** wireless sensor networks, hierarchical clustering, cooperative communication, energy conservation

## Abstract

In this work, we present an energy efficient hierarchical cooperative clustering scheme for wireless sensor networks. Communication cost is a crucial factor in depleting the energy of sensor nodes. In the proposed scheme, nodes cooperate to form clusters at each level of network hierarchy ensuring maximal coverage and minimal energy expenditure with relatively uniform distribution of load within the network. Performance is enhanced by cooperative multiple-input multiple-output (MIMO) communication ensuring energy efficiency for WSN deployments over large geographical areas. We test our scheme using TOSSIM and compare the proposed scheme with cooperative multiple-input multiple-output (CMIMO) clustering scheme and traditional multihop Single-Input-Single-Output (SISO) routing approach. Performance is evaluated on the basis of number of clusters, number of hops, energy consumption and network lifetime. Experimental results show significant energy conservation and increase in network lifetime as compared to existing schemes.

## 1. Introduction and Motivation

One of the main design challenges in wireless sensor networks (WSNs) is coping with resource constraints placed on individual sensor devices. One of the resource constraints to meet is power consumption. As physical size of a sensor node decreases, so does energy capacity [1]. Energy constraints end up creating limitations such as computational power and limited coverage that lead to architectural issues.

**Figure 1.**
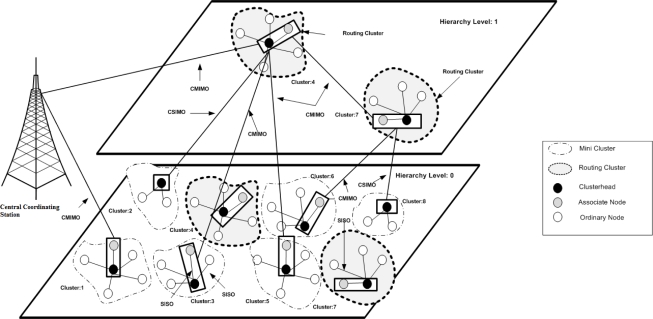
Hierarchical cooperative communication architecture.

In dense deployments spanning large areas, the sensor network usually has a mesh topological structure [2]. In such deployments, a number of sensor nodes route their messages via other nodes in the network. Some of the sensor nodes are configured to turn their power on and off (various power modes may be available) in order to minimize energy consumption. Therefore, when two neighboring sensors have to communicate, both the nodes must be in active (on) mode [2]. Clustering protocols help in achieving this objective in a scalable and efficient manner by organizing sensor nodes into small groups called cells or clusters. Communication cost is a crucial factor in deciding the network life time reflected by the number of messages passed among the neighboring nodes. In wireless sensor networks, clustering involves grouping sensor nodes and electing a clusterhead (CH) similar to having base transceiver stations (BTS) in cellular communication. Nodes within a cluster/cell can directly communicate with their CH. CHs forward aggregated data to the central coordinating station (CCS) directly or via multiple hops using other CHs as intermediate forwarding nodes. Thus, the collection of clusterheads in the network forms an overlay network of sensor nodes (see Figure 1 for architecture diagram). In single hop clustering algorithms, various studies make assumptions regarding direct communication between CHs to the CCS. Such assumptions may seem too strict for realistic situations as, in some cases, the CH may have limited transmission range.

Network longevity is one of the fundamental design objectives of wireless sensor networks [3]. In recent years, cooperative MIMO has been proposed as a communication model to be used in ad hoc wireless sensor networks [3–5]. Multi-input multi-output (MIMO) technology has the potential to increase channel capacity and reduce transmission energy consumption. Because of the requirement of energy efficiency in large networks, a concept known as virtual MIMO has attracted a growing interest. In virtual MIMO network, a group of sensor nodes cooperate to transmit and receive data. This technique is also known as cooperative MIMO (CMIMO). Participation of multiple transmitters and receivers in a transmission saves significant energy in long-range communications [6]. Energy can be conserved if we allow nodes to cooperatively transmit data just like in MIMO mode of communication. Due to circuitry complexity and difficulty of integrating separate antennas on sensor nodes, cooperative sensor nodes exploit virtual MIMO in wireless sensor networks for energy efficient communication and enhance data reliability [3].

Hierarchical routing (HR) is an efficient routing approach in wireless sensor networks. In hierarchical routing, for a given network setting, nodes are grouped into small clusters at base level 0, which are further grouped in to bigger clusters at level 1 and so on. This provides excellent scalability by providing a node state of O(logN) for routing and addressing [7].

In [8], Lee *et al*. discuss capacity scaling laws of wireless ad hoc networks using hierarchical cooperation. Their findings show an order optimal linear throughput scaling is achievable for some networks using hierarchical cooperation (HC).

Wireless sensor networks pose stringent constraints in terms of energy efficiency, network lifetime and data reliability. Most of these contributions are on theoretical level and to the best of our knowledge, no work exists in wireless sensor networks domain for hierarchical cooperation with virtual MIMO for ensuring energy efficiency in a real-life WSN setting.

### 1.1. Contributions

The main contributions of this work are as follow:

- A novel scheme for energy efficient hierarchical clustering to be used with cooperative MIMO communication for a wide area sensor coverage with higher network lifetimes in WSNs. The algorithm is distributed and scales to large networks.
- An energy efficient cooperative clusterhead selection algorithm which minimizes energy expenditure.
- Performance analysis of our scheme against prevalent schemes for cooperative MIMO. Performance is analyzed on the basis of extended network life time, energy conservation and minimization of number of hops from clusterhead (CH) to central coordinating station (CCS).

### 1.2. Organization

The remainder of the paper is organized as follows. Section 2 describes the previous work done in MIMO and wireless networks. We define the problem in Section 3. In Section 4, we explain our scheme, hierarchical cooperative clustering, whereas, in Section 5 we present our network model and experimental results. Section 6 summarizes our conclusions.

## 2. Related Work

In the past few years, MIMO has established itself as a reliable and energy conserving technology for wireless networks. Recently, MIMO has been proposed in conjunction with sensor networks to improve energy conservation, network throughput and reliability in fading channels [9]. In [9], the authors propose a multiple-input multiple-output (MIMO) technique where multiple nodes within a cluster cooperate in signal transmission and reception. A cross-layer design is used for efficient routing and minimal energy consumption and delay. For cooperative MIMO scheme, routing criterion is set based on an equivalent single-input single-output (SISO) system, where each cooperating cluster is treated as a super node.

In [5], authors present a detailed analysis of the dissipated power during a sensor node’s operation. Authors extend the work presented in [4] where they analyze the best modulation and transmission strategy to minimize total energy consumption required to send a given number of bits. It is shown that traditional belief of MIMO systems being more energy-efficient than SISO systems in Rayleigh fading channels may be misleading when both the transmission energy and the circuit energy consumption are considered. The paper demonstrate that in short-range applications, especially when the data rate and the modulation scheme are fixed, SISO systems may outperform MIMO systems as far as energy efficiency is concerned. Nevertheless, if constellation size is optimized then MIMO outperforms SISO even for short distances. In [5] the authors have implemented a simple cooperative node selection algorithm to achieve higher energy gains in the MIMO approach, and examined how their algorithm affects the calculated thresholds. Moreover, authors have reached expressions to estimate threshold values regarding the channel conditions, the distance between source and destination nodes, and the network density, which determine the areas where the MIMO structure is more energy-efficient. The authors show that if channel conditions are unfavorable, multihop SISO approach proves to be more energy-efficient. They further argue that as the network density increases, gains are achieved due to multihop transmission. However, for large networks, single hop MIMO may not be practical. This work does not cover clustering aspect in sensor networks. Clustering is important for scalability and offers a reduced communication flow which is important for saving energy.

In [3], authors propose a distributed MIMO-adaptive energy-efficient clustering/routing scheme, coined cooperative MIMO (CMIMO), which aims at reducing energy consumption in multihop WSNs. In CMIMO, each cluster has two cluster heads, which are responsible for routing traffic between clusters (*i.e.*, inter-cluster communications). CMIMO has the ability to adapt the transmission mode and transmission power on a per-packet basis. The transmission mode can be one of four transmit/receive configurations: 1 × 1 (SISO), 2 × 1 (MISO), 1 × 2 (SIMO), and 2 × 2 (MIMO). It employs a dynamic clustering approach with two clusterheads in a cluster. There is a master CH (MCH) which gathers data from the sensory nodes in the cluster while the second one is a slave CH (SCH). The two CHs operate as a cooperative MIMO node for inter-cluster communications. The operation of CMIMO has three main phases: cluster formation, intra-cluster communications, and inter-cluster communications with cooperative MIMO capabilities. In this scheme, reclustering affects the whole network, and not only the cluster to which the MCH belongs. The new selection of clusterheads affects the functions of the nodes, as an MCH may become an SCH or a non-CH node. Similarly, an SCH may also become an MCH. This change in nodes’ functions necessitates reclustering the whole network. Reclustering in CMIMO is an energy intensive task which in worst case may require the whole network to get reclustered. Further, there are no bounds on the number of cluster pairs in the network.

In [10], authors propose a multilayer hierarchical architecture to cover more sensing area and to distribute energy across the network. The work focusses on hierarchy creation in the network without incorporating benefits of a scheme like cooperative MIMO suitable for long-range wider area coverage. Further, various aspects such as cluster-head rotation and scaling with number of nodes have not been addressed.

Forero *et al*. [11] provide a clustering mechanism for spatially distributed data in wireless sensor networks using deterministic and probabilistic approaches to unsupervised learning. Again, the work is distinctive for its focus on distributed data aggregation and clustering than energy efficiency and long-range cooperative MIMO communication.

In this work, we propose an architecture for energy efficient clustering to be used with cooperative MIMO communication in WSNs. Our scheme is hierarchical, offers an efficient reclustering approach and scales to large networks. In subsequent sections, we provide details of proposed scheme and use hierarchical MIMO/hierarchical cooperative clustering interchangeably given our proposed framework attains benefits of both MIMO and cooperative clustering.

## 3. Problem Formulation

The main sources of energy consumption in sensor nodes are sensing data, processing data and communication. Generally, sensor nodes can operate in four modes: transmit, receive, idle and sleep. The highest power consumption is in the transmit mode and lowest in the sleep mode, whereas in idle mode nodes consume power almost equal to that of receive mode. In this section, we formulate the hierarchical cooperative MIMO clustering problem as an estimation of number of clusters which offers minimum energy consumption in the network. Let *E_total_* denote total energy consumption in our sensor network (Energy model is discussed in Section 4.6). The total energy is taken as sum of energy consumed in intra-cluster (*E_local_*) and inter-cluster communication (*E_CH_*).

(1) $$E_{\mathrm{total}}=E_{\mathrm{local}}+E_{\mathrm{CH}}$$

We assume sensor nodes having a uniform distribution in a square area of size 2*a* × 2*a*. Let *N* be the total number of sensor nodes in our network and *k* be the number of clusters in the cluster set *K*. There are *H* hierarchical levels. For the scope of our problem, the objective is to find number of clusters in the network which minimize energy expenditure for all set of nodes within the network. In this case, our objective function is to minimize total energy consumption subject to the condition that there are *k* clusters in the network, each node belongs to a cluster and the distance between a clusterhead and a member sensory node is less than the maximum transmission distance of the node. The first constraint ensures maximum connectivity in the network. Second constraint puts a restriction that all nodes in wireless sensor network should belong to at least one cluster. This is to maximize coverage. The third constraint restricts distance (d) between a CH and an ordinary sensor node (SN) to be less than the maximum transmission distance *d_tx_* of nodes. Once the number of clusters k^*^ is found, we determine number of backbone/routing clusters (RCHs) (Section 4.4) satisfying minimum total energy constraint within the network while ensuring *RCHs* ≤ *k*. For a fully connected graph, we aim at minimizing the total cost (energy expenditure). While doing so, the objectives are:

- Increased network lifetime.
- Minimum energy expended at each node.

## 4. Hierarchical Cooperative MIMO Clustering

In this section, we explain the proposed architectural scheme and the clustering/routing algorithm. We also provide a discussion on design considerations for the hierarchical cooperative clustering.

### 4.1. Architecture

The proposed hierarchical cooperative clustering algorithm selects nodes to forward data by employing MIMO capabilities. We explain the basic architecture with the help of Figure 1. In the figure, basic network components and node setting have following assumptions and meanings:

- **Node Spread:** A number of nodes are deployed on the grid shown as hierarchical level zero.
- **Cluster Formation:** At level zero, nodes are divided to form small clusters (cluster formation explained in Section 4.2). Each cluster is led by a node called the clusterhead. A few clusters have cooperative nodes as well.
- **Overlay Formation:** Once network is divided into clusters, clustering is re-triggered to form an overlay network on top of it. At level one of the hierarchy, two of the mini clusters act as routing clusters (RC). Routing clusters form a backbone for forwarding data to the CCS.
- **Cooperative MIMO:** A CH is responsible for aggregating data from nodes in the cluster. In the case when CH cannot send information directly to the CCS (energy/distance/link quality limitation), it forwards the data to cooperative node. This is to establish a set of transmitters for cooperative MIMO communication.
- **Intra-Cluster SISO Communication:** Cooperative communication is energy efficient for a certain threshold distance. Sensor nodes within a cluster are in close vicinity with clusterhead. Therefore, sensor nodes send data to clusterhead through SISO communication.
- **Clustering Parameters:** Clustering parameters include geographic location of nodes, neighbors list, residual energies, link quality (LQ) and boundary constraints on each of these parameters. LQ is incorporated as the receiver’s noise figure (Table 1) and Signal to Noise ratio against a desired bit error rate. The limit on number of clusters is a function of number of nodes in a cluster subject to minimum energy consumption in the cluster (limit on number of clusters is discussed in Section 4.6).

The data is relayed via cooperative communication to next hierarchical levels. The cardinality of cooperative communication is decided on the basis of transceiver distance. Figure 1 shows an example of a topology of a wireless sensor network with hierarchical cooperative clustering. The clusterhead and the cooperative node which lie at level zero of the hierarchy communicate with the routing clusters which are at level 1 of the network hierarchy.

Following is the basic clustering pseudocode and associated energy and design considerations.

**Table:** 

Hierarchical Cooperative Clustering: Pseudo Code and Design Considerations
**Input:** PeriodicTimer.fired()
Parameters: Noise Model, Transmission range, Clustering Parameters
**Method:** Clustering Nodes broadcast HELLO message to one hop neighbors. Nodes exchange neighbor information. Hierarchy Creation Based on clustering parameters nodes decide the CH. The CH decides cooperative node based on minimal energy consumption. Routing Clusterhead Selection CHs from neighboring clusters exchange ROUTE Message CHs select routing clusterheads (RCHs) from the existing set of clusterheads (CHs). CHs and RCHs decide communication mode. CHs periodically update their routing tables. Design Considerations Factors affecting clustering. Factors affecting routing. Factors affecting reclustering. Energy Model Based Cluster Sizing Determine total energy consumed for cluster formation using per bit energy in a cluster, energy for longhaul communication and local circuit energy (explained subsequently). Use first derivative method to find minima and number of clusters satisfying our constraints.

The pseudocode is elaborated further in subsections below.

### 4.2. Clustering

The basic clustering follows following steps.

#### 4.2.1. Information Exchange

Initially, when sensor nodes are placed, the network is not clustered. When a node wakes up and finds that its not a part of any cluster it triggers clustering algorithm. When the algorithm starts, nodes contend for the channel using carrier sense multiple access collision avoidance (CSMA/CA) scheme. Each node sends its neighbor node a HELLO message. The HELLO message is broadcast at a low power level. Aim of using low power is to send the message to only one hop neighbors which are in close geographic vicinity and to save power. The HELLO message consists of node ID, its remaining energy and a list of its neighbors. Every node calculates its weight on the basis of neighborhood information. Weight is the sum of the normalized values of clustering parameters. For implementation simplicity, we assume that a node’s weight is equal to its own residual energy (*i.e.*, the highest weight is assigned to residual energy of the nodes). Unlike LEACH algorithm [12] which assumes that each clusterhead has a radio powerful enough to directly reach the base station, in this work our focus is on sensor nodes spread over a large area where all nodes/clusterheads cannot communicate with CCS directly. In LEACH the clusterheads rotate after a given time and each node resumes the job of a clusterhead with probability p. In our work we designate the power of CH to the most suitable node and CH changes when the energy level drops below a certain level. This level is an arbitrary parameter and can be varied. Nodes continue to exchange this information for a finite number of rounds.

#### 4.2.2. Clusters Formation

Clusters formation consists of following steps:

**Step 1:** Each node compares its energy with that of its neighbors. If a node *v* has greatest amount of energy in its neighborhood, it declares itself a clusterhead. If there is a tie, it is resolved on the basis of node ID. Once a node declares itself a clusterhead, it propagates this message (“Cluster Formation”) to its next hop neighbors. Nodes which listen to this message become part of that cluster by sending a “Confirmation Message”. Once a node becomes a clusterhead or a part of a cluster, recurrent messages for clusterhead election are discarded by that node.
**Step 2:** If *v* is not the best node, it sends a “Clusterhead Message” to the node with the highest residual energy to become a clusterhead in case the best node has not already declared itself a clusterhead.
**Step 3:** After sending the “Clusterhead Message” to the best node, node *v* waits for a fixed duration of time (*δ*) for the best node to reply.

If that best node and not any other node in the neighborhood sends a “Cluster Formation” message, node *v* declares it self to be a clusterhead after a fixed time interval (*δ*) .

#### 4.2.3. Choosing the Cooperative Node (CN)

Choice of cooperative node is made in a way to possibly maximize the network lifetime. This is achieved depending upon the distance from CCS and the clusterhead. Cooperative nodes are chosen in such a way that they lie in close geographic vicinity of clusterhead. Second criteria in selection of CN is residual energy of the cooperative nodes. If the clusterhead can directly communicate with the CCS, no cooperative node is selected in the cluster. Such a case occurs when cluster and CCS lie at a small distance. In this case there is Single Input Multiple Output (SIMO) communication between the cluster and the CCS. This is illustrated in Figure 1. When both the clusterhead and cooperative nodes communicate with the CCS, the communication operates in MIMO mode. Cooperative node is selected each time clusterhead wants to send data. Clusterhead is aware of residual energies of nodes in the cluster. If a node lying in close geographic vicinity starts getting drained off, the clusterhead selects another node with a higher amount of residual energy.

### 4.3. Hierarchy Creation

Once the clustering process at level zero of hierarchy is completed, next step is selection of Routing Clusterheads (RCH). The neighboring clusterheads exchange a ROUTE message. In this message, clusterheads announce the cluster ID of the cluster whose clusterhead possesses the highest residual energy. Such a cluster becomes a routing cluster. The routing clusterheads announce their presence to all neighbor clusterheads in the network. The clusterheads update their routing tables. A RCH gets added in the routing table of a clusterhead if it takes less hops than the clusterhead itself to reach the CCS.

### 4.4. Routing Clusterhead Selection

Routing clusterheads (RCHs) collect data from different clusters (via any of the MIMO modes), aggregate that data and send it forward to the next RCH in the routing table. For their own cluster, they act as normal CHs with additional responsibilities of aggregation and forwarding for CH data in addition to their own data. Selection of cooperative nodes and participation in cooperative communication for RCHs follow same rules as CHs. Routing clusterheads carry the highest amount of energy, and provide maximum connectivity. Selection of routing clusterheads is based on energy efficiency and continues across layers if the next layer of clusterheads offer more energy conservation. For routing clusterhead selection, we need to find best links between the nodes to ensure reliable energy efficient data transfer. This asks for careful selection of clusterheads. A systematic procedure follows below.

We first construct our notation:

- *c_ij_* is a boolean variable which indicates a link between node *i* and node *j*. Two nodes have a link between them if they are in each other’s transmission range.
- *Q_ij_* is the weighted cost for sending an *L* bits message from node *i* to node *j*. Since, the goal is to minimize energy expenditure therefore cost *Q_ij_* is measured in terms of energy expended.

We use a weighted approach to determine the cost of each link.

(2) $$\min\sum_{i\in I}\;\sum_{j\in J}\;Q_{\mathrm{ij}}\;c_{\mathrm{ij}}$$

Subject to:

(3) $$p\leq \sum_{i\in I}\;c_{\mathrm{ij}}\leq q$$

where *i* is the clusterhead and *j* a sensory (ordinary) node. *p* and *q* are boundary constraints on the number of nodes in a cluster.

Weighted cost for all the links in the network is given by:

$$\left(\begin{array}{ccccc} Q_{11} & Q_{12} & \ldots & \ldots & Q_{1n}\\ Q_{21} & Q_{22} & \ldots & \ldots & Q_{2n}\\ \ldots & \ldots & \ldots & \ldots & \ldots \\ \ldots & \ldots & \ldots & \ldots & \ldots \\ Q_{n1} & Q_{n2} & \ldots & \ldots & Q_{\mathrm{nn}} \end{array}\right)$$

The cost of a link is determined by the following factors:

1. Transmission distance: Neighboring clusters determine links between each other on the basis of allowed transmission distance. If the distance between node i and j is greater than the allowed transmission distance, the link *c_ij_* is assigned 0, otherwise link *c_ij_* is assigned 1.
2. Energy: Once links between the clusterheads are decided, the clusterheads start clustering algorithm to elect a routing clusterhead. The neighboring clusterheads exchange a ROUTE message. In this message, clusterheads announce the cluster ID of the cluster whose clusterhead possesses the highest residual energy. Only the clusterheads take part in this process and not the cooperating nodes since clusterheads are default communication centers with all the required knowledge about sensory nodes in clusters.
3. Geographic distance: A RCH gets added in the routing table of a clusterhead if it takes less hops than the clusterhead itself to reach the BS.

Based on the above three metrics, each cluster announces its cost *Q_i...n_*. Cluster with minimum cost becomes the routing clusterhead. Routing clusterheads announce their presence to all neighbor clusterheads in the network. The clusterheads update their routing tables.

At the end of clustering, each node is either a clusterhead, a routing clusterhead or an ordinary node affiliated with a clusterhead. Thus the network is fully connected, except for the case where the network gets partitioned in a way such that no two border nodes are in the communication range of each other. Thus our algorithm satisfies the *ad hoc clustering properties* mentioned in [13]. Our algorithm is distributed because every node has knowledge about its one hop neighbors only. Similarly, routing clusters are selected by triggering clustering algorithm at a distance of one hop from each clusterhead.

### 4.5. Design Considerations

In this section, we discuss factors affecting clustering, routing and reclustering significance for the design of Hierarchical MIMO.

#### 4.5.1. Factors Affecting Clustering

There are three main factors which affect the clustering process. The first is the transmission range of a sensory node. A node is able to communicate with any other node within a given transmission range. As we increase transmission range, there is also an increase in the radius of a cluster. For sparse networks, we may have to increase the transmission range but for dense networks the transmission range is kept less so as not to burden the clusterheads. The second parameter is the residual energies of nodes. A node with a higher amount of energy is a better candidate for becoming a clusterhead. The third factor is link quality.

#### 4.5.2. Factors Affecting Routing

**Figure 2.**
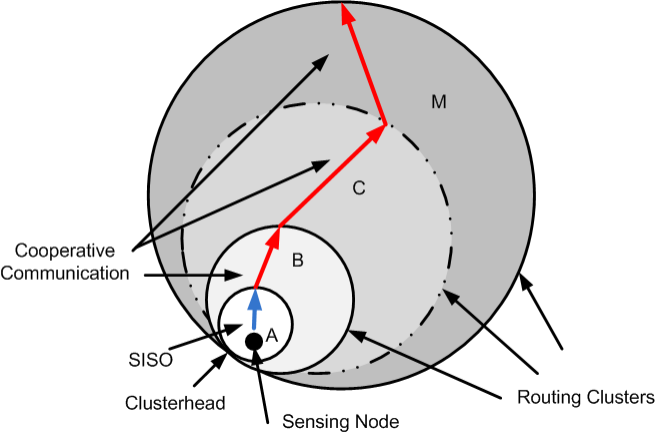
Hierarchical cooperative routing.

In the proposed scheme, we forward data to the CCS via hierarchical routing (Figure 2). The main aim of hierarchical routing is to efficiently preserve the energy of sensor nodes by involving them in multihop communication and performing early data aggregation in order to decrease the number of transmitted messages to the CCS. Each cluster is associated with a routing cluster. When a cluster gets associated with a routing cluster, it calculates the amount of energy required to send data to the routing cluster. It then send a PING message to CCS. If cost of sending PING message is higher or if CCS is not in the transmission range, cluster adds its routing cluster as next hop “node” in its routing table. In case CCS is in the close vicinity of the cluster, CCS becomes next hop neighbour.

#### 4.5.3. Factors Affecting Reclustering

Topology maintenance is critical for a clustered network. There are certain cases in which an update is required in a cluster structure. This happens in any of the following four scenarios:

- When a node *v* is powered on, it broadcasts a HELLO message to all its neighbors to determine if there is a clusterhead in the neighborhood. If there is a clusterhead, the node joins the clusterhead. Otherwise, it compares its energy with the energies of its neighbors to see if it has the highest energy in its neighborhood. If so it becomes a clusterhead. If not, it asks the node with highest energy to become clusterhead.
- A clustering update is required when a link is created between two nodes.
- When an existing link is broken:
  – If one node is a clusterhead and the other is an ordinary sensory node then both will trigger clustering again.
  – If both the nodes are ordinary nodes then both nodes announce their new weights to their clusterheads and all the neighbors. This would make the clusterheads update the neighbor list of their neighbors.
- When the residual energy of a clusterhead reaches a threshold value (an energy parameter), clusterhead delegates the responsibility to another node in the cluster who has high enough energy to become a clusterhead. If some of the neighbors of old clusterhead cannot communicate with the new clusterhead then either they re-trigger clustering or join neighboring clusters. This property of our algorithm overcomes the flaw in state-of-the-art CMIMO [3] where in worst case the whole network has to get reclustered. In our setup when energy of a clusterhead reaches one third of its initial energy (when it initially became a CH), it designates the responsibility to another node with a higher amount of energy (subject to availability of such a node in the cluster).

### 4.6. Energy Model Based Cluster Sizing

In this section, we use an underlying communication energy model to determine the number of clusters (and thus, the cluster sizes). Using knowledge of the network under consideration, we find number of clusters required for minimum energy expenditure within the network. In Section 5, we compare the number of clusters based on energy model with the results we achieve using the proposed distributed HMIMO scheme.

Let *d_u,v_* be the average inter-cluster distance. Expected distance between cluster/node *u* and cluster/node *v* located at coordinates (*x_i_*, *y_i_*) and (*x_j_*, *y_j_*) is given by [14]:

(4) $$m[d_{u,v}]=\int \int_{R}\;\sqrt{x^{2}+y^{2}}\;\frac{1}{4a}\;\mathrm{dxdy}$$

where *x* = *x_i_* − *x_j_*, *y* = *y_i_* − *y_j_* and the length of one side of the grid is 2*a*.

We assume distant dependent Friss free-space model (*d*^2^ power loss) [15] for per node energy consumption. Thus energy consumption in a sensor node for transmitting one bit of information over a distance *d* is:

(5) $$E_{\mathrm{local}}=E_{\mathrm{circuit}}+{ɛ}_{f\;s}\;{\left(d_{\mathrm{toCH}}^{*}\right)}^{2}$$

The energy required to transmit one bit from the clusterhead to the cooperating node is also equal to Equation (5). Since the CCS is far from sensors, presumably the energy consumption follows multi-path model. Therefore, assuming *k* clusters and *N*/*k* nodes in a cluster, energy per bit consumed in a cluster is given by [15]:

(6) $$E_{\mathrm{CH}}=(\frac{N}{k}-1)\;E_{\mathrm{circuit}}+\frac{N}{k}\;E_{\mathrm{DA}}+E_{\mathrm{circuit}}+{ɛ}_{\mathrm{mp}}\;{\left(d_{u,v}^{*}\right)}^{4}$$

where *E_DA_* is the energy consumed in data aggregation and *E_circuit_* is [3]:

(7) $$\begin{array}{l} M_{t}\;(P_{\mathrm{DAC}}+P_{\mathrm{mix}}+P_{\mathrm{filt}})+2P_{\mathrm{syn}}+\\ \;\;\;\;\;\;\;\;\;M_{r}\;(P_{\mathrm{LNA}}+P_{\mathrm{mix}}+P_{\mathrm{IFA}}+P_{\mathrm{filr}}+P_{\mathrm{ADC}}) \end{array}$$

and ${ɛ}_{\mathrm{mp}}\;{\left(d_{u,v}^{*}\right)}^{4}$ is given by:

(8) $$\left(1+\alpha )\;(\gamma (M_{t},\;M_{r})\;N_{o}\;B\;N_{f}\;G_{o}\;M_{l}\;{\left(d_{u,v}^{*}\right)}^{4}\right)$$

where *γ*(*M_t_*,*M_r_*) is the required SNR at the receiver. *N*_0_ is the power spectral density, *B* is the bandwidth, *N_f_* is the receiver’s noise figure, *G*_0_ is antenna gain and *M_l_* is the link margin. *M_t_* and *M_r_* is the number of transmitters and receivers respectively. For the case where we have single input and single output at the transmitter and receiver ends, *M_t_* and *M_r_* are both one and P_*_ are power consumption values.

We first find number of hops in a given network setting. Assume a uniform distribution of clusters in a given network. Assuming each side of the square field has $2a/\sqrt{k}$ clusters, number of hops (h) in the network is approximately given by:

(9) $$h=\frac{k+\sqrt{k}k}{2}$$

From Equations (1) and (9), we derive the expression for total energy consumption in the network for *L* bits.

(10) $$E_{\mathrm{SISO}}=(E_{\mathrm{CH}}+(\frac{N}{k^{*}}-1)\;E_{\mathrm{local}})\;L.h$$

where k^*^ from [15] is:

(11) $$k^{*}=\frac{\sqrt{0.5855\;N\;{ɛ}_{f\;s}\;a^{2}}}{{ɛ}_{\mathrm{mp}}\;{\left(d_{u,v}^{*}\right)}^{4}-E_{\mathrm{circuit}}}$$

Equation (11) gives the number of clusters in the network for multihop traditional SISO communication [15] (for minimum energy expenditure). We construct an overlay network on top of these clusters. The bottom layer communicates with the top layer in MIMO mode. Number of clusters in overlay network which minimizes the total energy consumption as compared to when data is forwarded via multihop SISO is given by:

(12) $$E_{\mathrm{total}}\;(k_{h})<E_{\mathrm{total}}\;(k_{\left(h-1\right)})$$

where *h* indicates the hierarchical level. Energy consumed in MIMO communication (*E_MIMO_*) is the sum of local energy consumption incurred in local communication between the clusterhead and the cooperating node and the energy consumed in the long haul communication.

(13) $$E_{\mathrm{MIMO}}=E_{\mathrm{local}}+E_{\mathrm{CH}}+E_{\mathrm{longhaul}}$$

From Equation (10), energy consumption in a clustered structure is given by:

(14) $$E_{\mathrm{total}}=(E_{\mathrm{CH}}+E_{\mathrm{longhaul}}+(\frac{N}{k}-1)E_{\mathrm{local}})\;\mathrm{Lh}$$

For long haul communication clusterhead to clusterhead distance or clusterhead to CCS distance is very large. The communication follows a multipath model. Thus, assuming:

(15) $$D_{u,v}>>d_{u,v}$$

*E_longhaul_* is a function of distance *D* which includes energy required for data transfer from clusterhead to cooperative node, and data transfer from cooperative node to next hop cluster. First term in Equation (16) indicates circuit energy consumption at the cooperative node, second term indicates transmission energy consumption for local communication and last term indicates energy consumed in long haul communication.

(16) $$E_{\mathrm{longhaul}}=3E_{\mathrm{circuit}}+{ɛ}_{f\;s}\;{\left(d_{\mathrm{toCH}}^{*}\right)}^{2}+3{ɛ}_{f\;s}\;{\left(D_{u,v}^{*}\right)}^{4}$$

From Equation (14), we intend to minimize the total energy spent in intercluster communication. By means of first derivative method to find minima, we differentiate Equation (16) w.r.t *k*, and get an expression for number of routing clusterheads (k^*^) for cooperative communication, which minimizes total energy.

(17) $$k_{\mathrm{RCH}}^{*}=\sqrt{\frac{0.1951\;k^{*}\;{ɛ}_{f\;s}\;a^{2}}{{ɛ}_{\mathrm{mp}}\;{\left(D_{u,v}^{*}\right)}^{4}-E_{\mathrm{circuit}}}}$$

## 5. Evaluation

In this section, we provide the network model used to conduct validation of our approach. We provide the results achieved and a discussion on the results. We test the proposed scheme on TOSSIM over TinyOS 2.x. Synchronization between nodes is provided by TOSSIM’s default stack [16].

### 5.1. Network Model

The nodes in our network model have unique IDs and have location coordinates namely *x* and *y* deployed using a uniform random distribution. Initially, all the nodes have same amount of energy. Two nodes are called neighbors if they are within the transmission range of each other. All the results have been averaged on 100 different runs for varying distribution of nodes.

For the energy analysis, we consider communication on Rayleigh fading channel. We take optimum constellation sizes for MIMO and SISO communication, same as suggested by [4]. All other values are assumed as mentioned in Table 1, otherwise stated. All nodes generate packets independently. We have used poisson distribution (*λ*) for packets generation per 10 seconds.

We assume sink node to be located outside the square field (sensor nodes deployment) unless stated otherwise. The CCS is equipped with multiple antennas. Multi-hop operation based on an energy-efficient routing is used for intercluster communications. We assume signal to noise ratio (SNR) required to achieve a BER of 10^−3^ based on [3]: 24.4 dB for SISO, 10.6 dB for SIMO, 14.1 dB for MISO, and 6.9 dB for MIMO. For performance analysis we implement and compare our scheme with CMIMO [3] and traditional multihop SISO approach.

### 5.2. Results and Discussion

#### 5.2.1. Number of Clusterheads

**Figure 3.**
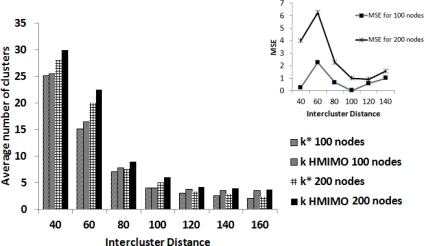
Number of clusters.

In this section, we compare our simulation results with the theoretical bound we achieved using a centralized clustering approach. In practice, using a centralized approach may not be feasible in large sensor networks due to amount of information required regarding state of the network. Our algorithm uses a distributed approach to form clusters aiming to provide a close enough approximation to performance in theory. In Figure 3, through simulations, we compare the performance achieved in proposed HMIMO scheme with theoretical results. We use mean square error (MSE) as a metric to compare the number of clusterheads arrived at through proposed algorithm with the number of clusterheads derived in Equation (17). Our results suggest that, for a given network setting (various instances of uniformly distributed nodes), number of clusters with minimum energy expenditure is close to the values derived in Equation (17). We plot a graph for N = 100 and N = 200 nodes. Figure 3 shows that for 100 nodes, when intercluster range is between 80 m and 120 m, MSE is less than 1, showing that our algorithm performs close to theory. As the network becomes more and more dense, the value of MSE also increases as compared to a sparser network setting, a factor to be kept in consideration to reap benefits of MIMO in an actual network deployment.

In subsequent experiments, we show that the overall energy consumption of our proposed scheme is less than the previously proposed methods. Since the overall energy consumption in the network and network lifetime are dependent upon the underlying clustering scheme, therefore, through this set of simulations, it is established that the proposed clustering scheme provides results close to our theoretical analysis.

#### 5.2.2. Energy Consumption

**Figure 4.**
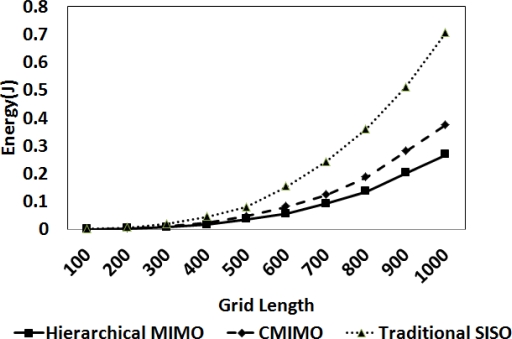
Energy (J) comparison for three algorithms.

**Figure 5.**
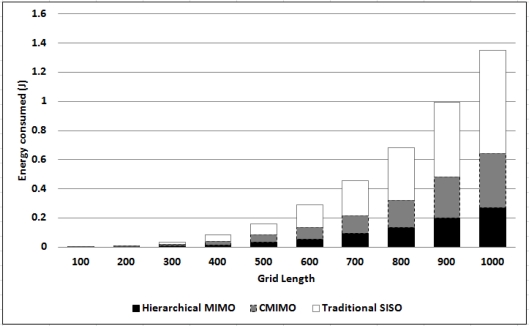
Energy (J) consumption per bit.

For energy consumption of HMIMO, we consider 100 nodes randomly deployed within a square region. Figure 4 shows a comparison between hierarchical MIMO, CMIMO and traditional SISO. For this set of experiments, we tested our algorithm for a grid size from 100 × 100 to 1, 000 × 1, 000 and intercluster range taken as 33% of the grid size. In hierarchical MIMO, data is forwarded via hierarchical routing. We use cost based link state routing for the other two algorithms. We verify our results for a path loss exponent between 2.7 (semi-furnished rooms) and 3 (densely furnished buildings) [17]. For smaller grid sizes, performance of all the three algorithms is similar. This is because the intracluster distance between the clusterhead and sensory nodes and the intercluster distances between the communicating clusterheads is less. For shorter distances, the proposed algorithm and CMIMO use SISO mode of communication. As we increase the grid size, traditional multihop SISO continues to provide communication via SISO approach. However, other two algorithms start switching to cooperative modes. In such scenarios, HMIMO outperforms CMIMO since number of hops for the data to reach CCS are less in HMIMO. The reason for decrease in number of hops is the hierarchical nature of the proposed algorithm. As we increase the network area, there is an upto 15%–20% energy saving for HMIMO compared to CMIMO. In another setting, we set the intercluster range to be 25% of the grid size. This is well depicted in the graph in Figure 5. Proposed HMIMO outperforms the traditional approaches even for large intercluster distance. This is because at large inter-cluster distance, clusters use MIMO communication in most cases which is energy conservant communication mode.

In state-of-the-art CMIMO (described in Section 2), as the intra-cluster range (range among individual clusters) increases, fewer clusters are formed. Thus, transmission distances between CHs become larger and circuit energy becomes less significant than transmission energy, which results in making modes other than SISO more favorable. A decrease in number of clusters increases number of nodes in each cluster which over-burdens the clusterheads which are the communication centers. In our scheme, clustering parameters are not changed for the purpose of saving energy in routing. Distance dependent routing takes place by selecting appropriate nodes as routing clusterheads with the purpose of energy conservation. The advantage of our scheme is that routing and clustering jointly conserve power.

#### 5.2.3. Network Lifetime

**Figure 6.**
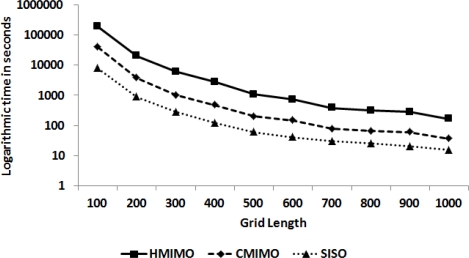
Comparison of network lifetime.

In this experiment, we measure life time of the network under consideration. We calculate network lifetime based on how much a node is used for communication. We analyze this performance metric on the basis of an energy parameter (an arbitrary threshold value). We measure and compare the algorithms depending upon the time when 50% of nodes reach this threshold value. Figure 6 shows a comparative analysis. Network employing hierarchical cooperative clustering has a longer life time as compared to CMIMO and SISO (When comparing the algorithms, distribution of nodes and packets generation is the same). An improvement in energy conservation at the communicating clusters elongated network lifetime manifolds. There is initially an increase in network lifetime with an increase in grid length because the intercluster distance increases, which makes MIMO a more favorable communication mode. Nevertheless, when grid length is stretched beyond 500 m, the total number of hops also increases, which results in a decrease in network lifetime for our algorithm as well as CMIMO.

**Figure 7.**
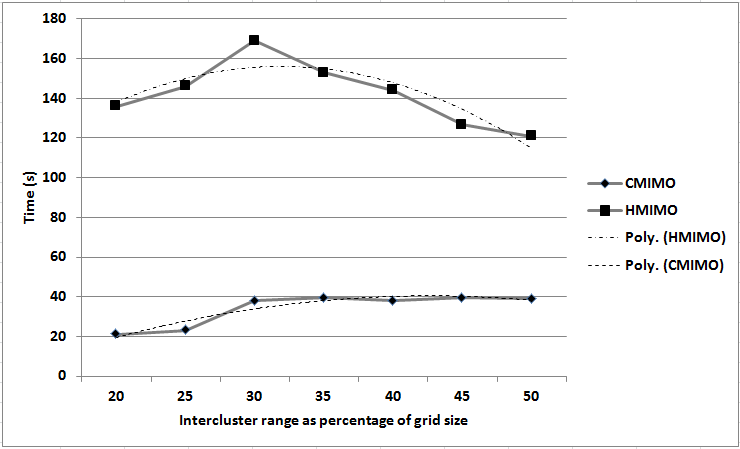
Network lifetime for various intercluster ranges.

Figure 7 shows a comparison of the time taken for networks employing HMIMO and CMIMO to reach an energy threshold value. The graph is plotted for intercluster range as the percentage of grid size versus time. In this experiment grid size is set to 1,000 with CCS in the centre of the square grid. The two dotted lines are the trend lines for HMIMO and CMIMO. For shorter distances, SISO mode of communication is prevalent in both the algorithms. As the intercluster distance increases, clusterheads switch to MIMO mode of communication which is depicted in the graph by an increase in network lifetime. MIMO outperforms SISO for a certain distance range. When grid size is approaching 1,000, there is drop in network life time for both CMIMO and our algorithm.

#### 5.2.4. Transmission Modes

**Figure 8.**
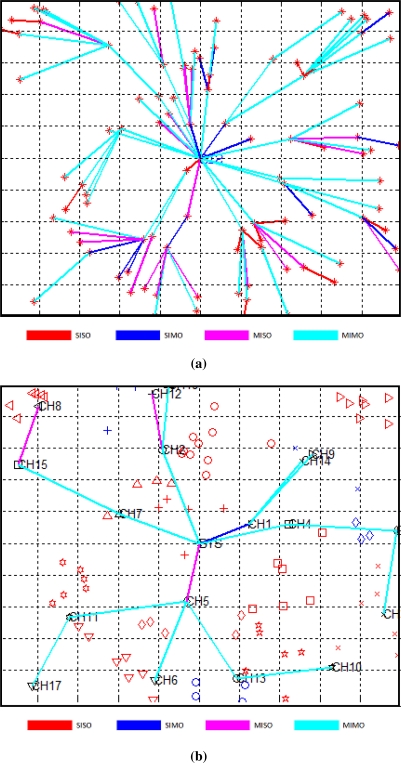
Comparison of Routing Nodes in CMIMO and HMIMO. **(a)** Routing in CMIMO; **(b)** Routing in HMIMO via backbone Clusterheads.

Figure 8 shows a topology of our network (*k* = 100). In this figure, CCS lies at the center of square grid. The four different colored links show communication modes between clusters. Figure 8(a) shows various communication modes for routing in CMIMO. The diamonds, stars, etc., in the same neighborhood show nodes belonging to same cluster in Figure 8(a). In Figure 8(b), packets are routed via HMIMO. There are less number of hops in hierarchical cooperative communication as compared to the traditional CMIMO approach. The figure shows that MIMO mode of communication is dominant in the network.

#### 5.2.5. Number of Hops

**Figure 9.**
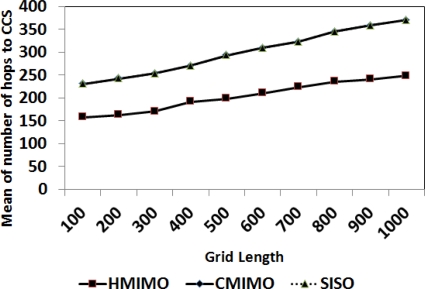
Mean number of hops to Central Coordination Station.

In this experiment we study the impact of grid size on the number of hops required for data to reach the CCS. Figure 9 shows the comparison of the impact of different grid size ranges on number of hops. The graph is plotted as length of grid versus number of hops. As the grid size increases the transmission distance is increased, hence number of hops between sensory nodes and CCS increases. Note that the number of hops in CMIMO and traditional SISO remain the same (The two curves are overlapping). HMIMO has an edge over the previously proposed mechanisms in terms of reduction in number of hops.

**Figure 10.**
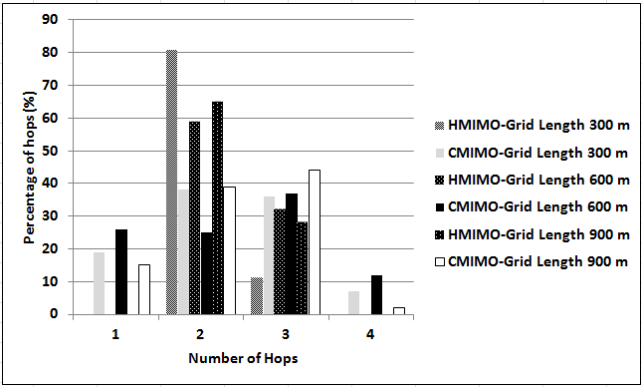
Histogram of number of hops to Central Coordination Station.

Figure 10 shows the percentage comparison of number of hops for our scheme and in CMIMO. For a grid size of 300 × 300, data is reaching CCS 80% of the time in two hops. For large networks, the number of hops in CMIMO are increasing, whereas there is no node that requires more than three hops to send data to CCS for this particular network setup and parameters.

## 6. Conclusions

In this paper we presented an architectural scheme for cooperative clustering which employs hierarchical routing featuring cooperative communication among the sensor nodes. The novelty of our scheme lies in the fact that our scheme saves energy by addressing three different critical design considerations. Firstly, as compared to state-of-the-art CMIMO, our clustering algorithm does not necessitates clustering of the whole network in case the responsibility of clusterhead has to be shifted to another node. Secondly, the clustering algorithm also results in a near optimal number of clusters in the network, a property that state-of-the-art CMIMO lacks. Thirdly, by employing cooperative MIMO in hierarchical routing, our algorithm gives better performance in comparison to existing routing techniques. Most of the time, clusters use 2 × 2 MIMO for communication. The clustering algorithm selects clusters on the basis of four factors, namely: geographic location, link quality, residual energy of nodes and boundary constraints on the number of nodes in a cluster. Each cluster is led by a clusterhead which selects the cooperative node such that the energy required to communicate with the cooperative node is minimum. As circuit energy dominates the transmission cost for shorter distances, therefore, a node in a close geographic vicinity of the clusterhead is selected as the cooperative node. Experimental results show 15%–20% energy gain as compared to CMIMO and more than 50% energy savings as compared to traditional SISO for large distances. Network lifetime also shows a significant increase when compared with CMIMO. Number of hops to CCS in hierarchical MIMO also shows improvement as compared to previously proposed schemes.

Since the underlying clustering scheme is distributed, performance in terms of energy expenditure can be further improved by using a centralized clustering approach for cooperative communication. However, this may not be practical since nodes may not have complete knowledge of the network. Secondly, even if nodes acquire this knowledge through exchange of messages among each other, the cost incurred in exchange of such messages, cluster formation and reclustering in a centralized clustering approach may surpass the energy conserved during cooperative communication of data.

Considering implementation in real life scenarios, a few applications that can benefit from our proposed scheme can be monitoring of mines and oil and gas installations. Since our scheme gives improved results for large networks, we believe that implementing this scheme on real sensors for detecting and reporting faults and anomalies in above applications can be highly effective. This work lays foundations for such implementations while ensuring energy efficiency and enhanced network lifetime. Our subsequent investigations in WSN domain focus on utilizing the proposed scheme for such applications.

## Figures and Tables

**Table 1.** System Parameters.

**Parameter**	**Meaning**	**Value**
*R_b_*	Bit Rate	1 Mbps
*P_DAC_*	Digital-to-Analog converter	15 mW
*P_ADC_*	Analog-to-Digital converter	15 mW
*P_mix_*	Mixer	30.3 mW
*P_filt_*	Active filters at transmitter	2.5 mW
*P_filr_*	Active filters at receiver	2.5 mW
*P_syn_*	Frequency synthesizer	50 mW
*P_LNA_*	Low noise amplifier	20 mW
*P_IFA_*	Intermediate frequency amplifier	2 mW
*B*	Bandwidth	10 KHz
*N*_0_	PSD	−171 dBm/Hz
*M_l_*, *N_f_*	Link margin, Receiver noise figure	10 dB
